# P-929. Impact of Infectious Diseases Consultation on Antimicrobial Stewardship and Clinical Outcomes: A Prospective Cohort Study

**DOI:** 10.1093/ofid/ofaf695.1133

**Published:** 2026-01-11

**Authors:** Hemanth H, Suresh Kumar Dorairajan, Sherlin M S

**Affiliations:** Sri venkateswara college of pharmacy, Chennai, Tamil Nadu, India; Apollo hospitals,Vanagaram, Chennai, Tamil Nadu, India; Sri venkateswara college of pharmacy, Chennai, Tamil Nadu, India

## Abstract

**Background:**

Antimicrobial stewardship is essential to optimize antibiotic use, reduce resistance, and improve clinical outcomes. Involvement of Infectious Diseases (ID) specialists has been shown to enhance stewardship efforts. This study compares antibiotic utilization patterns, stewardship interventions, infection types, and microbiological profiles among hospitalized patients managed with versus without ID consultation.

**Figure:**
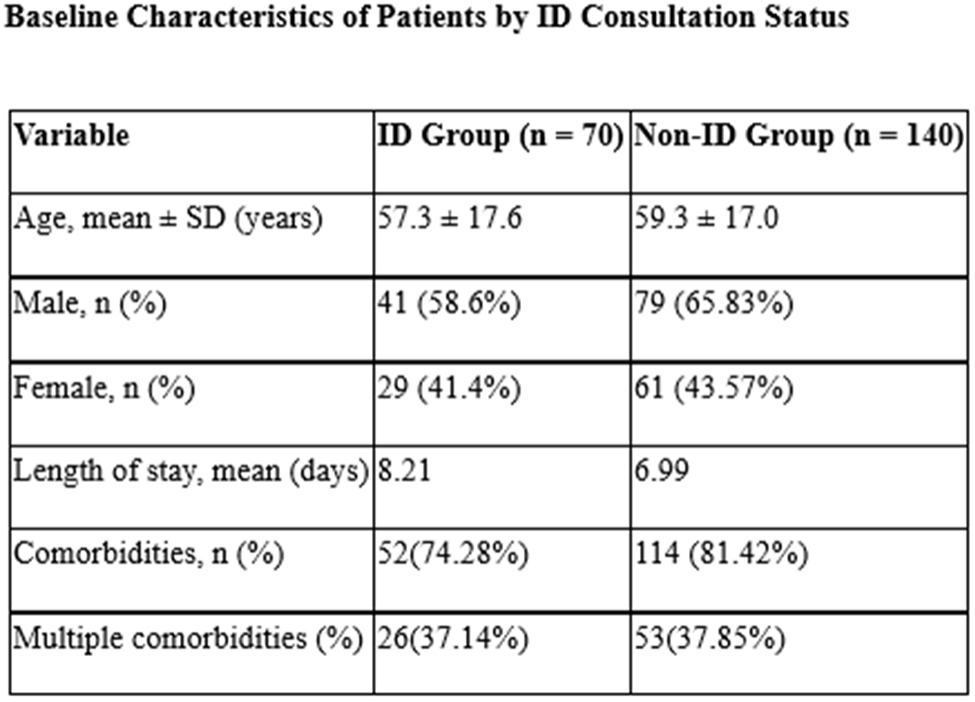

**Figure:**
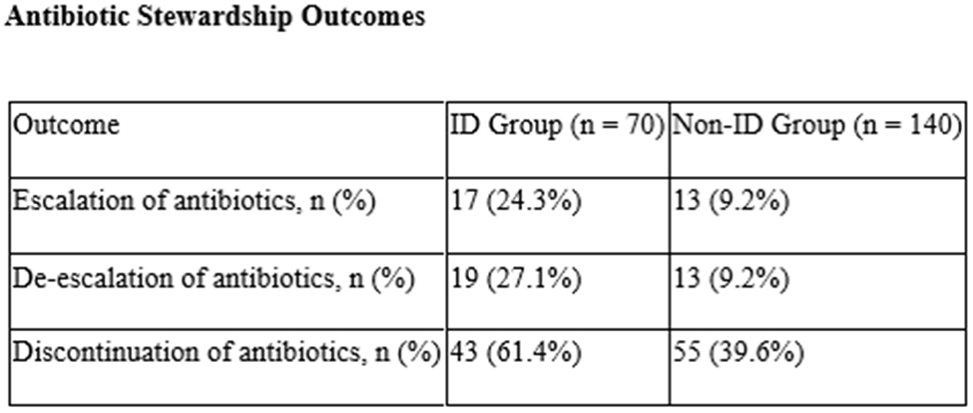

**Methods:**

A prospective observational cohort study was conducted at a tertiary care hospital between October 2024 and April 2025. A total of 210 adult in patients who received systemic antibiotics for > 48 hours due to confirmed or suspected infections were enrolled. Of these, 70 patients received ID consultation, while 140 did not. Patient demographics, infection categories, empirical antibiotic use, stewardship measures (escalation, de-escalation, discontinuation), microbiological isolates, and resistance patterns were collected and analyzed.

**Figure:**
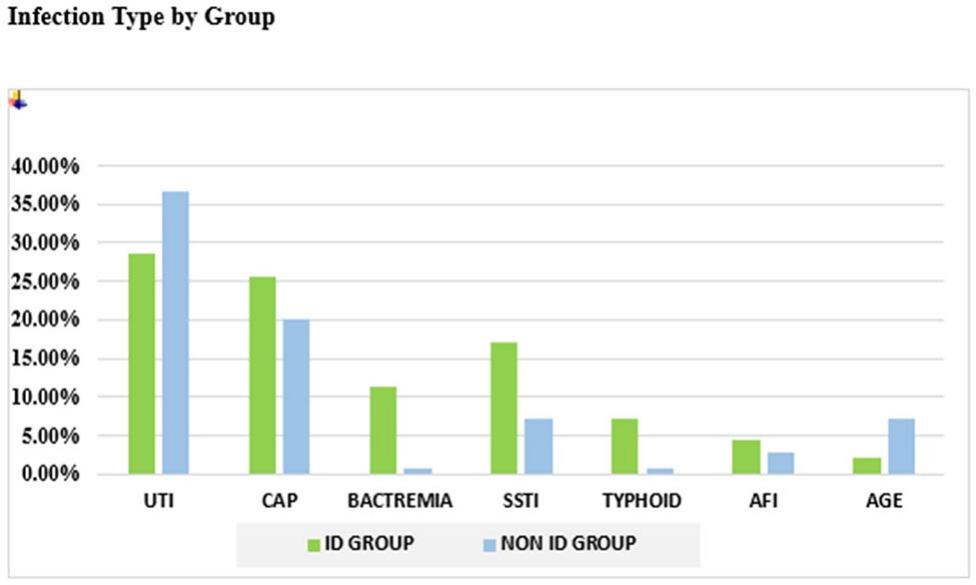

**Figure:**
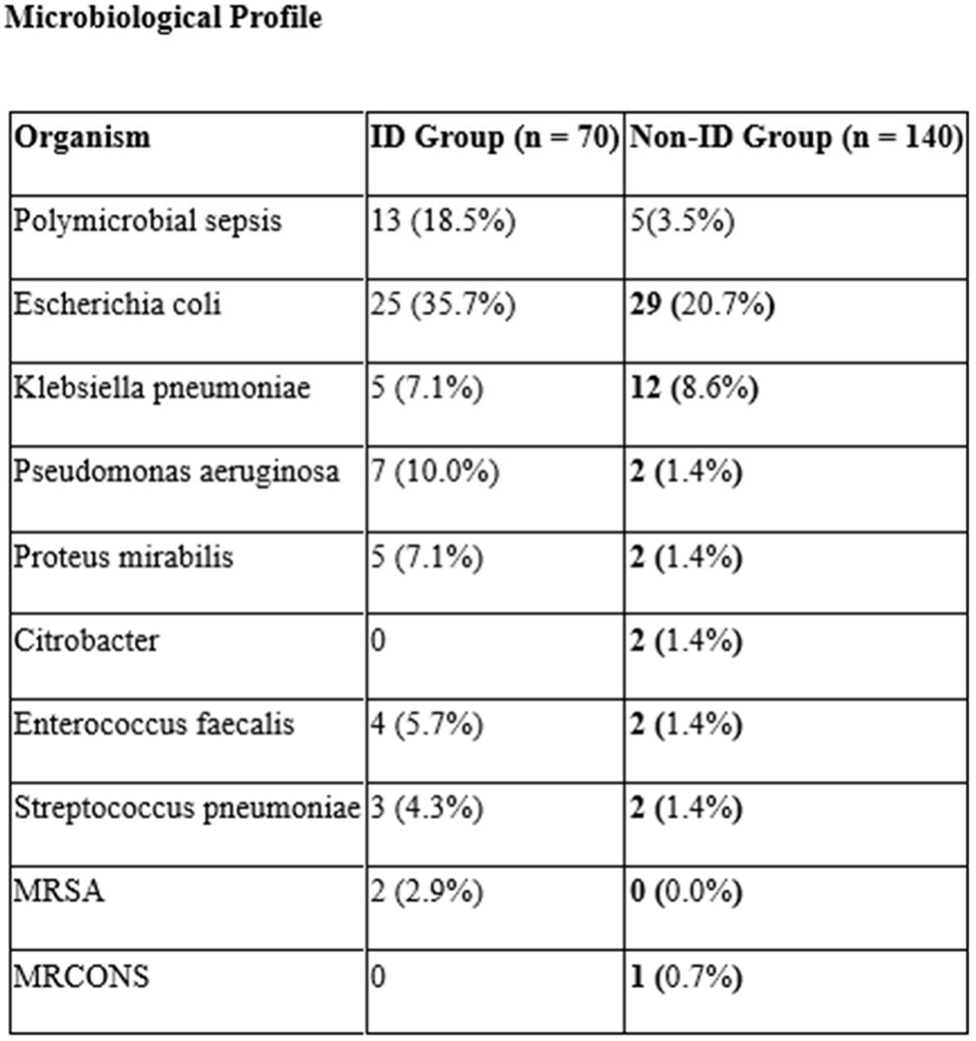

**Results:**

Of 210 patients, 70 received ID consultation. The mean age was 57.3 ± 17.6 years in the ID group and 59.3 ± 17.0 years in the non-ID group. Other Baseline characteristics were similar. The ID group had longer stay (8.21 vs. 6.99 days) and more narrow-spectrum (55.7% vs. 46.4%) and multiple antibiotics (77.1% vs. 66.4%). Stewardship outcomes were more frequent in the ID group: escalation (24.3% vs. 9.2%), de-escalation (27.1% vs. 9.2%), and discontinuation (61.4% vs. 39.6%). Microbiological diagnosis was more robust in the ID group, with higher detection of polymicrobial sepsis (18.5% vs. 3.5%). Bacteremia, SSTIs, typhoid was higher in the ID group; dengue and scrub typhus were more common in non-ID. Microbiological yield and pan-sensitive isolates were higher with ID input, though carbapenem resistance was also more frequent (14.3% vs. 6.4%).

**Conclusion:**

ID specialist consultation was associated with improved antimicrobial stewardship practices, higher diagnostic yield, and better antibiotic optimization. These findings highlight the critical role of ID physicians in inpatient care and support broader integration of ID expertise to enhance stewardship and combat antimicrobial resistance.

**Disclosures:**

All Authors: No reported disclosures

